# Treatment of High-Grade Chronic Osteomyelitis and Nonunions with PerOssal^®^: A Retrospective Analysis of Clinical Efficacy and Patient Perspectives

**DOI:** 10.3390/jcm13247764

**Published:** 2024-12-19

**Authors:** Jonas Armbruster, Florian Bussmann, Holger Freischmidt, Gregor Reiter, Paul Alfred Gruetzner, Jan Siad El Barbari

**Affiliations:** BG Klinik Ludwigshafen, Department for Orthopedics and Trauma Surgery, Clinic at Heidelberg University, Ludwig-Guttmann-Str. 13, 67071 Ludwigshafen, Germany; jonas.armbruster@bgu-ludwigshafen.de (J.A.);

**Keywords:** bone defects, nonunion, chronic osteomyelitis, PerOssal^®^, bone graft substitute, lower extremity functional scale

## Abstract

**Background/Objectives**: Traditional autologous bone grafts as a treatment for bone defects have drawbacks like donor-site morbidity and limited supply. PerOssal^®^, a ceramic bone substitute, may overcome those drawbacks and could offer additional benefits like prolonged, local antibiotic release. This study investigates the clinical and radiological outcomes, including patient-reported outcomes, of using PerOssal^®^ in nonunions (NU) and high-grade chronic osteomyelitis (COM). **Methods**: A single-center, retrospective study, investigating patients treated with PerOssal^®^ between January 2020 and December 2023. Collected data include patient characteristics as well as various surgical and outcome parameters including the Lower Extremity Functional Scale (LEFS). **Results**: A total of 82 patients were analyzed. Reinfection occurred in 19.5% of cases. Osseous integration of PerOssal^®^ was achieved in 89% of cases, higher in cavitary defects (91.5%) than segmental defects (72.7%). The revision rate was 32.9%, mainly due to wound healing disorders and reinfections. Mean LEFS score was 53.4 which was heavily influenced by sex (male: 50.7 vs. female: 63.4), revision surgery (no: 55.7 vs. yes: 49.1), reinfection (no: 56.6 vs. yes: 39.4), and osseous integration of PerOssal^®^ (yes: 55.8 vs. no: 38.4). **Conclusions**: PerOssal^®^ demonstrates promising outcomes in treating NUs and high-grade COM, especially in cavitary defects, with high osseous integration rates and acceptable functional results. However, reinfection remains a concern, particularly with difficult-to-treat pathogens and extensive surgical histories. Early, comprehensive surgical intervention and tailored antibiotic strategies are essential. Patient selection, defect characteristics, and comorbidities significantly influence success. Further research is needed to optimize treatment protocols.

## 1. Introduction

The treatment of bone defects remains a major challenge in orthopedics and traumatology [1,2]. These defects often result from the surgical treatment nonunions (NU), whether aseptic (ANU) or septic (SNU), or from chronic osteomyelitis (COM).

Despite varying definitions, a nonunion is generally diagnosed when a fracture fails to heal without additional medical intervention [3]. Despite advances in stabilization techniques, nonunions occur in 10–30% of fractures, particularly in complex long-bone fractures of the tibia [4]. Consequently, nonunions represent a major complication in trauma and orthopedic surgery. The socioeconomic burden is immense, with treatment costs multiplying compared to those fractures that heal promptly and without adverse sequelae at the same location [5].

The occurrence of osteomyelitis (OM), an inflammation of the bone marrow and bone tissue (osteitis), continues to pose a challenging problem in orthopedic and trauma surgery [6]. While aseptic osteomyelitis exists [7], the primary cause is either a local bacterial infection, e.g., due to complex fractures or infected nonunions or the systemic spread of infection to the bone, with *Staphylococcus aureus* as the predominant pathogen [8]. Despite advances in surgical antisepsis and treatments such as antibiotic-loaded allogenic bone grafts, the prevalence of OM continues to rise, particularly among older patients, largely due to age-related immune system decline [9,10].

Chronic conditions such as diabetes and chronic kidney disease (CKD) further complicate the treatment and outcomes of NU and OM. Diabetes is associated with impaired bone metabolism and delayed fracture healing due to chronic hyperglycemia, microvascular complications, and an increased risk of infection [11,12]. Similarly, CKD adversely affects bone remodeling through a range of mechanisms, including mineral and hormonal imbalances, which collectively contribute to compromised fracture healing and increased susceptibility to infection [13].

Effective treatment of NU and OM is composed of a dual approach, involving radical surgical debridement of all infected and necrotic tissue, accompanied by systemic antibiotics [14]. The importance of surgical intervention is emphasized by the fact that local avascularity, as found in bone sequesters, can hinder the effectiveness of systemic antibiotics. Since surgical debridement often leads to significant bone defects, a two-stage treatment approach is often necessary. After successful debridement, the bone void is filled with an antibiotic-loaded polymethylmethacrylate (PMMA) spacer [15]. In a second-stage surgery the bone defect then needs to be filled [15,16].

Different defect sizes require different approaches to optimize healing. Small bone defects (<5 cm) can be filled with non-vascularized bone grafts, such as autologous cancellous bone or allografts. Larger bone defects are typically treated with long bone segment transport or vascularized bone grafts [17,18].

The current gold standard for treating smaller bone defects is autologous transplantation of iliac crest bone graft material [19]. Although healing rates are acceptable, this procedure is associated with donor-site morbidity and limited by the patient’s available bone resources [19,20]. As a result, allogenic bone grafts (allografts) have been a focus of research for a considerable time [21].

The ideal bone graft substitute should have antimicrobial, osteoinductive, and osteoconductive properties [16,22,23,24]. Antibacterial effects can be achieved by creating a basic milieu such as in bioactive glasses [25], or by preloading microporous ceramic-based bone graft substitutes with antibiotics [26,27], which leads to a higher local concentration of these antibiotics with few systemic side effects [22]. Such localized antibiotic delivery has demonstrated efficacy against difficult-to-treat pathogens, including methicillin-resistant *Staphylococcus aureus* (MRSA) and multidrug-resistant Gram-negative bacteria (MDRGN) [28]. Osteoinduction refers to the stimulation of undifferentiated mesenchymal stem cells to differentiate into osteoprogenitor cells and produce new bone [29]. Osteoconduction is defined as the process of the ingrowth of cells from a surrounding bone bed into the porous structure of an implant [29].

PerOssal^®^ (Osartis GmbH, Münster, Germany) is a synthetic, biodegradable, and osteoconductive bone substitute material composed of 51.5% nanocrystalline hydroxyapatite and 48.5% calcium sulfate [30]. While calcium sulfate acts as a growth factor and is mostly resorbed within 6 weeks, hydroxyapatite is resorbed more slowly, providing a scaffold for bone ingrowth (osteoconductivity) [22,31,32]. PerOssal^®^ is available in pellet form (6 × 6 mm) and can be loaded with various antibiotics, such as Gentamicin or Vancomycin [27]. Compared to traditional carriers like collagen sponges [33], the microporous structure of PerOssal^®^ enables a prolonged, sustained release of the antibiotics [27]. Unlike other ceramic-based bone grafts, where antibiotics are impregnated intraoperatively [22], PerOssal^®^ is hardened before antibiotic loading, leading to fewer complications such as the drainage of liquid bone graft residues [23].

The clinical use of PerOssal^®^ in patients has been reported in a limited number of publications. Berner et al. first described its successful use in treating a tsunami victim with lower limb OM in 2008 [34]. Subsequently, PerOssal^®^ was used in 19 patients with spondylodiscitis, with promising results, by von Stechow et al. [32]. Further studies followed, including a single-center study in Italy that treated 52 patients with mostly limited-stage chronic osteomyelitis [35]. Sambri et al. first published on the treatment of 13 patients with fracture-related infection of the distal tibia using a combined orthoplastic approach, meaning all patients had their soft tissue defect covered with a free flap [36]. They later published the largest cohort to date, comprising 93 patients treated for chronic osteomyelitis [37].

The patient cohort analyzed in the present study differs in key characteristics from those in the aforementioned studies, particularly in terms of OM progression, age, and pathogen spectrum (see Section 4 for details). Notably, previous studies have not yet assessed subjective outcomes or patient satisfaction after treatment with PerOssal^®^, such as through patient-reported outcome measures (PROMs).

This study aims to address this gap by investigating the clinical (both subjective and objective) and radiological outcomes following the use of PerOssal^®^ in NU and high-grade COM, with particular attention paid to patient-reported outcomes. Additionally, it seeks to identify specific subgroups for which PerOssal^®^ may be particularly beneficial by analyzing specific patient characteristics, such as the number of previous surgeries, the size and characteristics of the bone defect (cavitary vs. segmental), and the type of pathogen (difficult-to-treat or not). See Section 2 for more details about all the collected parameters.

Through this, the authors aim to test the hypothesis that the treatment of NU and COM with PerOssal^®^ has reinfection, revision, and integration rates comparable to those of autografts, as well as comparable or better patient satisfaction.

## 2. Materials and Methods

The study was approved by the ethics committee of the Medical Association of Rhineland-Palatinate, Germany (2022-16749_2).

This single-center, retrospective cohort study included patients treated between January 2020 and December 2023 with PerOssal^®^ for either COM or NU. The follow-up period was at least 6 months post-surgery. Table 1 summarizes the inclusion and exclusion criteria.

After identifying patients based on the inclusion and exclusion criteria (Table 1), letters were sent to obtain written consent at least six months after the index surgery. Patients were also asked to complete the Lower Extremity Functional Scale (LEFS) in its German version. The LEFS is a validated instrument that assesses an individual’s ability to perform 20 daily activities, each graded on a scale from 0 to 4, resulting in a total score ranging from 0 to 80, with higher scores indicating better function [38,39]. All patients were additionally contacted once by phone to inquire about any revision surgeries, verify their medical history, and identify any adverse events that had occurred since their last appointment at the implanting clinic. The timing of the LEFS assessment therefore corresponds to the end of the follow-up period.

All patients underwent multiple infection marker tests, X-rays and at least one preoperative MRI scan. These diagnostic tools were used to categorize SNU, ANU, or COM. SNU and COM were diagnosed using published criteria for fracture-related infections. If patients did not meet these criteria, NU were classified as aseptic. No other indications for PerOssal^®^ existed during the study period. COM was further classified with the Cierny–Mader classification according to the consensus document for the diagnosis of peripheral bone infection in adults [40].

Surgical management was conducted accordingly to previously described protocols [18,41,42]. Patients were treated either with a one-stage or a two-stage protocol. Step 1 surgery began with a radical surgical debridement, including the excision of all necrotic tissue (both bone and soft tissue) until viable tissue was exposed. At least 5 samples were collected for microbiological and histological testing, as recommended in the literature [43]. When the clinical and intraoperative impression were leading to an aseptic nonunion or bone defect, step 2 was performed directly. If not, a PMMA spacer was inserted. The second surgery was then performed 6 weeks after the first.

Step 2 began with removal of the PMMA spacer and gathering of more microbiological samples if applicable. In either way, PerOssal^®^ was used according to the manufacturer’s instructions to fill up the defect after antibiotic loading in respect to the antibiogram of the causative pathogen. In case no antibiogram was available, Gentamycin was used to load PerOssal^®^ due to its broader antibiotic spectrum also covering gram negative bacteria. If necessary, bone defects were additionally filled with autografts or allografts on the surgeons’ discretion. Soft-tissue reconstruction by local or free microvascular muscle flaps was performed if soft-tissue coverage of the affected bone appeared insufficient and primary skin closure could not be achieved.

Antibiotic management: Perioperative, systemic antibiotics were administered according to the local microbiologic milieu if no causative pathogen was known. Most of the times this was performed with cefuroxime and clindamycin. When the microbiological culture was positive, the antibiotics were then changed to a more specific substance. If possible, biofilm-active substances (rifampicin, fosfomycin) were administered after wound secretion had stopped. Antibiotic treatment was then administered for 6–12 weeks based on the final microbiological results. In case of aseptic nonunion, antibiotic treatment was stopped after the final results of the microbiological testing came back negative.

Patient compliance was primarily monitored through regular follow-up visits in our outpatient department. Instances of non-compliance were addressed directly with the patients to encourage adherence to postoperative care protocols.

Table 2 summarizes the collected parameters.

OM is classified using the established Cierny–Mader classification, which classifies post-traumatic OM based on the extent of osseous involvement (Type I: medullary osteomyelitis; Type II: superficial osteomyelitis; Type III: localized osteomyelitis; Type IV: diffuse osteomyelitis) and physiological class (Type A: good immune system and delivery; Type B: compromised locally (B^1^) or systemically (B^2^); Type C: requires suppressive or no treatment, minimal disability, treatment worse than disease, not a surgical candidate) [44].

Integration of PerOssal^®^ in cavitary defects and consolidation of nonunions was evaluated in standard post-operative biplanar X-ray controls. Controls were continued until either integration or consolidation were present or revision surgery was necessary. The first image where integration or consolidation were stated by a fully blinded observer was taken as the integration or consolidation timepoint (refer to Section 4.5 for limitations; see Supplementary Materials Figure S2 for X-ray examples). The estimated volume of PerOssal^®^ used to fill the bone defect was determined based on the packages billed per patient. However, it is uncertain whether the entire amount was actually utilized.

Statistical analysis: Data were collected using Microsoft Excel (Microsoft Corporation, Redmond, WA, USA) and analyzed with SPSS Statistics v25 (IBM, SSPS Inc., Chicago, IL, USA).

The Kolmogorov–Smirnov test was employed to evaluate for normal distribution. Levene’s test was used to check for equal variances. To test for the statistical significance of differences between *n* > 2 parametric variables, one-way analysis of variance (ANOVA) followed by Bonferroni’s multiple comparisons test were performed. Two parametric, normal distributed variables were compared using Student’s independent t-test. Equal variances were assumed depending on Levene’s test. Non-parametric variables (ASA-Score, Cierny–Mader Classification) or parametric variables without normal distribution were compared using the Mann–Whitney U-test when split up in 2 groups or the Kruskal–Wallis-test for >2 groups. Comparison of non-parametric, nominal scale level variables was undertaken with the chi-squared test when applicable or Fisher’s exact test when one or more cells had an expected count of less than 5. Linear regression and bivariate correlations were used to determine variables with the most influence on subjective outcome. The Kaplan–Meier survival function was used to estimate PerOssal^®^ survival until revision was necessary. Patients were censored at the last available follow-up. A *p*-value < 0.05 was considered significant.

## 3. Results

### 3.1. Patient Characteristics

Between January 2020 and December 2023, 87 patients were treated with PerOssal^®^ for either NU or COM. Further analysis was conducted on 82 patients (94.3%) after excluding 5 patients (5.7%) who were lost to follow-up. These patients did not meet the minimum follow-up period of 180 days due to missed appointments at the implanting clinic and failure to respond to written or oral inquiries.

Table 3 shows the patient characteristics. Women were significantly younger than men (56.0 ± 1.9 years vs. 47.5 ± 3.8 years. *p* = 0.045) and had fewer pre-existing illnesses like high blood pressure or chronic kidney disease. Consistent with this, women had fewer cases of wound healing disorder.

In total, 4 patients died during follow up. All had a reinfection. Before revision surgery one patient died. Three had revision surgery. Of those, two had a reinfection, although sufficient integration of PerOssal^®^ was present.

Antibiotic loading was mostly performed with Gentamicin (58.5%), Vancomycin (20.7%), or both of them (11.0%). Other antibiotics used were Clindamycin, Tobramycin, and Meronem (9.8%).

No allergies or implant failures were observed. Table 4 shows the pathogens at the index surgery.

### 3.2. Patient Reported Outcome

**Figure 1:**
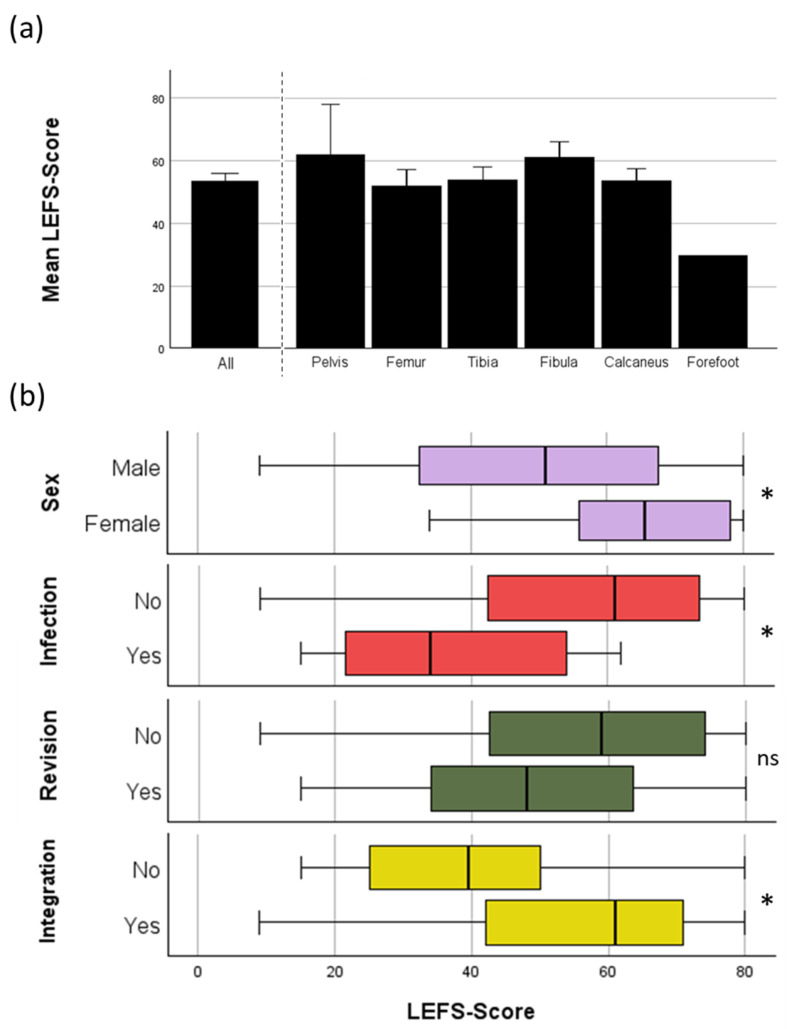
LEFS Outcome measurement: (**a**) mean LEFS score for all localizations (53.4 ± 2.5) and specific localizations of the lower extremity or pelvis. No statistically significant difference was observed; (**b**) analysis for main drivers for worse LEFS via linear regression showed highest differences in dependence on sex, infection, revision, and failed integration. Accordingly, direct comparison of LEFS showed significantly worse LEFS scores for male patients, patients who had reinfection, or in whom integration of the bone substitute failed. Revision in general lowered the LEFS score but statistical analysis remained non-significant. Medians are the black horizontal lines; interquartile range is the height of the rectangle; minimum and maximum value are the whiskers. LEFS: Lower Extremity Functional Scale; ns = not significant, * = *p* < 0.05.

LEFS was analyzed to evaluate patient reported outcome. In total, complete evaluation with the LEFS was possible for 66/82 patients (80,5%). Failure to use the LEFS came from either the usage of PerOssal^®^ in upper extremity (6/82 patients) or a missing response to the sent letter (11/82 patients). For the other outcome measurements, such as reinfection, revision, and osseous integration rates, those patients were further included as long as their follow-up time still met the criteria (see Section 2). The mean LEFS score was 53.4 ± 2.5 (see Figure 1a). Factors influencing the LEFS score included sex (male: 50.7 ± 2.8 vs. female: 63.4 ± 4.2), revision surgery (no: 55.7 ± 3.1 vs. yes: 49.1 ± 3.9), reinfection (no: 56.6 ± 2.6 vs. yes: 39.4 ± 4.9), and osseous integration of PerOssal^®^ (yes: 55.8 ± 2.5 vs. no: 38.4 ± 6.8). Women had a significantly better outcome compared to men.

Figure 1 shows the results.

### 3.3. Analysis of Reinfection

**Figure 2:**
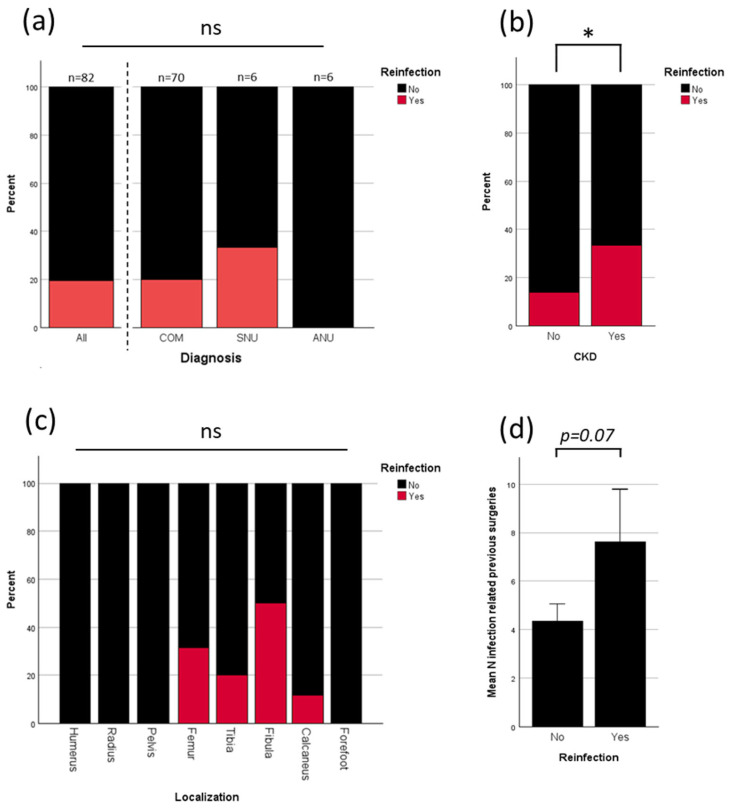
Analysis of reinfection: (**a**) percentage of reinfection in general and split up in between different initial diagnoses; (**b**) influence of chronic kidney disease on reinfection rate; (**c**) infection rate in different localizations; (**d**) mean previous surgeries in patients without and with reinfection. CKD is chronic kidney disease, COM is chronic osteomyelitis, SNU is septic nonunion, ANU is aseptic nonunion; ns = not significant, * = *p* < 0.05.

To evaluate the main drivers for infection, further analysis was performed. Overall reinfection rate was 19.5%. All reinfections were seen in patients who were treated for COM or SNU. No reinfection occurred in patients who were treated for ANU (Figure 2a). Patients with chronic kidney disease (CKD) had a significantly higher percentage of reinfection (Figure 2b). Although statistical testing showed no significant correlation between reinfection and localization, notably, all reinfections occurred in lower limbs (Figure 2c). Patients with more previous surgeries due to infection had a higher risk of further reinfection (*p* = 0.07; Figure 2d). Figure 2 shows the results.

There was no correlation between reinfection and other different preexisting conditions (diabetes mellitus, high blood pressure, coronary heart disease, peripheral artery disease, smoking) or the ASA-Score (Supplementary Materials Figure S1).

### 3.4. Analysis of Revision

**Figure 3:**
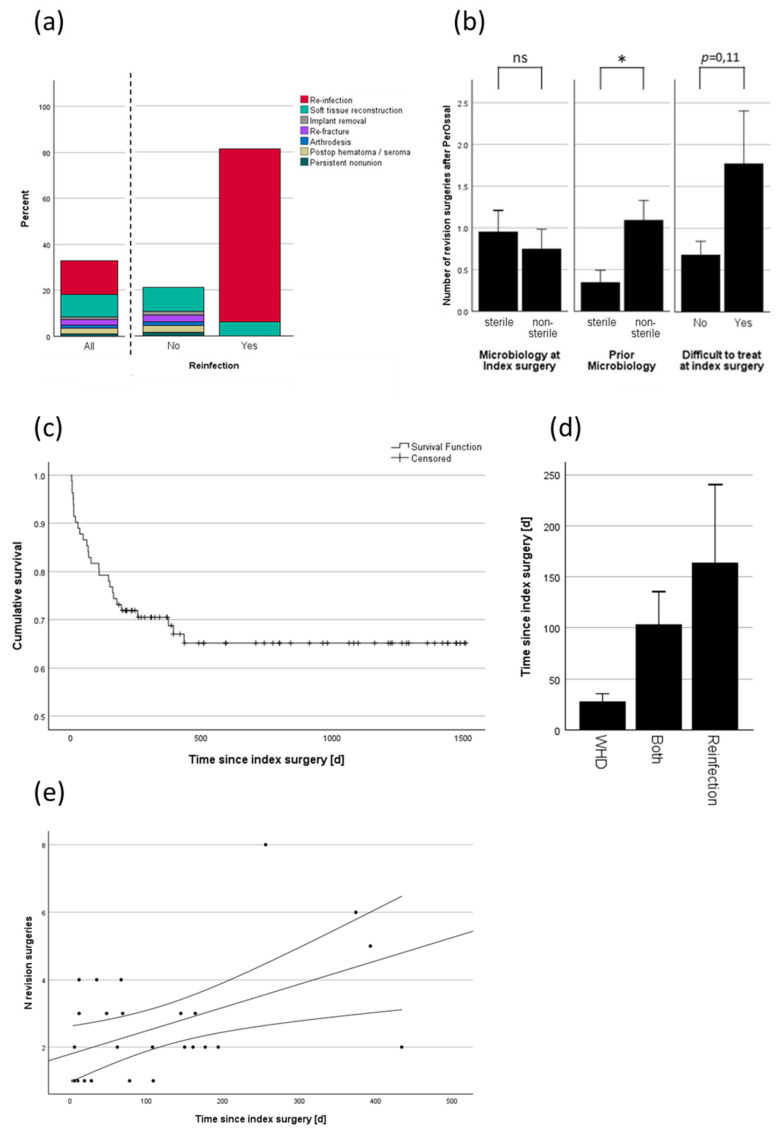
Analysis of revision: (**a**) percentage breakdown of reasons for revision surgeries in general and categorized by the presence or absence of reinfection; (**b**) impact of bacterial testing on revision rate; (**c**) Kaplan–Meier survival analysis of PerOssal^®^; (**d**) average time between index surgery and first revision for different complications; (**e**) correlation between the number of revision surgeries and the time between the index surgery and the first revision. Black lines indicate linear regression with 95% confidence intervals. WHD is wound healing disorder; ns = not significant; * = *p* < 0.05.

At the end of the follow-up period, the overall revision rate was 32.9% meaning a survival rate free from revision of 67.1% (Figure 3a). The primary reasons for revision surgery were wound healing disorders (WHD) or reinfections. Reinfection led to revision surgery in 81.3% of cases. Three patients had reinfection, but no revision surgery was performed. The reasons included death of the patient due to non-infectious cause, acceptance of a chronic, stable fistula, and rejection of revision surgery by the patient (Figure 3a). Microbiological parameters showed no effect on whether revision was necessary or not but on the amount of revision surgeries needed. Bacterial infection with prior positive microbiological testing was a major contributor to an increased number of revision surgeries (Figure 3b). The microbiological test result from the index surgery alone did not predict the need for revision surgery, but the presence of a difficult-to-treat pathogen showed a trend toward a threefold increase in the mean number of revision surgeries (Figure 3b). Figure 3c illustrates the survival function of PerOssal until the first revision, if revision was necessary. The mean time until first revision was 116 ± 23.8 days (mean ± SEM), with a median time of 69 (IQR: 152) days. The function indicates two distinct decreases in survival: one within the first two months, where revisions were primarily due to wound healing disorders, and another after 0.5–1.5 years, corresponding to revisions due to reinfection (Figure 3d). Wound healing disorders and reinfection can be interdependent, sometimes leading to an overlap in revision criteria. When both diagnoses were present, the time to revision averaged between the mean times associated with each condition alone (Figure 3d). There was a significant correlation (Pearson’s r = 0.5; *p* = 0.008) between the number of revision surgeries and the timing of the first revision surgery (Figure 3e).

### 3.5. Analysis of PerOssal^®^ Integration and Bony Consolidation of Nonunions

**Figure 4:**
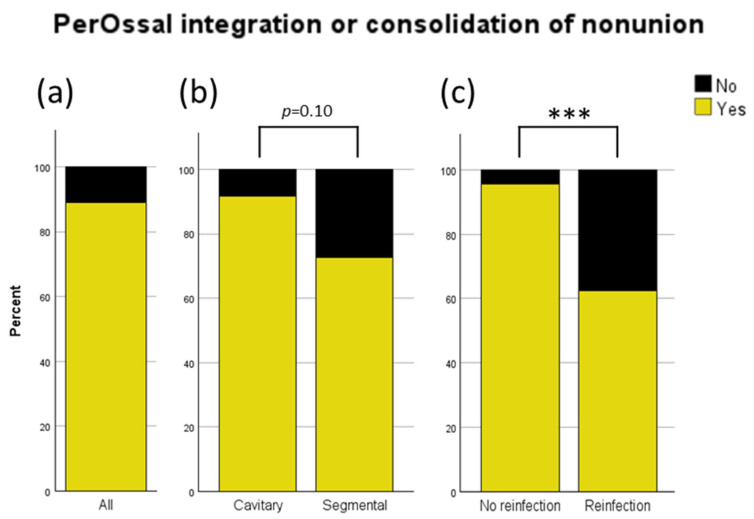
Analysis of integration of PerOssal^®^ in cavitary defects or consolidation of nonunions after usage of PerOssal^®^ in segmental defects: (**a**) overall percentage across all analyzed patients; (**b**) percentage in cavitary compared to segmental defects; (**c**) percentage in patients with and without reinfection. *** = *p* < 0.001.

The overall integration rate of PerOssal^®^ in cavitary defects or consolidation rate of nonunions after the use of PerOssal^®^ was 89% (Figure 4a). When analyzed by defect characteristics, the integration rate was even higher for cavitary defects, at 91.5%, compared to a consolidation rate of 72.7% for nonunions (Figure 4b; *p* = 0.10). Reinfection resulted in a significant decrease in the integration or consolidation rate (no reinfection 95.5% vs. reinfection 62.5%; Figure 4c). No significant differences were observed in integration time among SNU, ANU, and COM.

## 4. Discussion

### 4.1. Principial Findings

To our knowledge, this is the first study to analyze patient-reported outcome measures following the use of PerOssal^®^ and the second-largest patient cohort published to date [31].

The present study examines a cohort of 82 patients with a mean age of 54.3 years. Notably, female patients—constituting 20.7% of the cohort—were on average 8.5 years younger than male patients and had fewer pre-existing conditions such as high blood pressure or chronic kidney disease. Consistent with these observations, women experienced fewer cases of wound healing disorders, which may partly explain their better Lower Extremity Functional Scale (LEFS) scores (see Figure 1b). There were no gender-related differences in diagnosis, localization, or defect size. A total of 98.6% of patients presented with a grade III or higher Cierny–Mader classification, underlining that patients in this cohort were already in the advanced stage of the disease. PerOssal^®^ was predominantly used (over 90%) in lower extremity bones, with the tibia being the most common site. In approximately 70% of cases, Gentamicin was the selected loading antibiotic. The time interval between the initial fracture—if a fracture was part of the disease etiology—and the index surgery was 13.9 years. In cases of COM and SNU, the interval between the initial mention of infection and the index surgery was 8.9 years. On average, patients had undergone a total of 8.3 previous surgeries, both infection- and non-infection-related. These numbers highlight the high disease burden, the prolonged duration of patient suffering, and the complexity of cases treated in this study. Microbiological testing primarily showed a variety of different germs, but in 44.7% of patients a difficult-to-treat pathogen was identified. The mean defect size was 4.7 cm, which was later filled using primarily 50 (46.3%) or 12 (30.5%) bead packages of PerOssal^®^.

Notably, the minimal clinically important difference (MCID) for the LEFS is reported to be nine [45], indicating that the observed differences for sex, reinfection, and failed integration (see Figure 1) are clinically significant, as they exceed this threshold and significantly reduce the LEFS score. The effect of revision surgery showed a trend but did not reach statistical significance. Revision surgery, reinfection, and osseous integration are, of course, interrelated factors, making it difficult to differentiate the true effect of each individually.

Reinfection occurred in 19.5% (16/82) of all cases and in 20.0% (14/70) of COM cases. No infections were observed in ANU cases. Reinfection significantly lowered the rate of osseous integration (see Figure 4c). Only three of the sixteen patients with reinfection did not undergo revision surgery due to patient-specific factors (see Section 3.4), resulting in a revision rate of 81.3%, compared to an overall revision rate of 32.9%, and the rate of 21% in patients without reinfection, primarily due to necessary soft tissue reconstruction (see Figure 3a). All reinfections occurred in the lower extremity (Figure 3c), were significantly correlated with the presence of CKD (Figure 3b), and influenced by the number of prior surgeries due to past infections (Figure 3d). Reinfection rates did not differ significantly based on age, diagnosis in case of infection (SNU, COM), defect size, or defect characteristics (see Figure 3a and Supplementary Materials Figure S1). Patients with diabetes mellitus and a higher ASA score showed an increase in reinfection rates which remained not significant (see Supplementary Materials Figure S1).

In depth analysis of the revision rate revealed a significant correlation between the number of revision surgeries and the microbiological findings. However, it was not the microbiological results from the index surgery itself that significantly influenced the number of revision surgeries (sterile 0.95 ± 0.28 vs. non-sterile 0.75 ± 0.23), but rather the presence or absence of a DTT pathogen (no 0.68 ± 0.16 vs. yes 1.77 ± 0.63) or positive prior microbiological testing (sterile 0.35 ±0.15 vs. non-sterile 1.09 ± 0.24). One explanation could be that the cumulative prior microbiological testing may have been more sensitive than the microbiological testing conducted during the single index surgery, despite five samples being taken as recommended by clinical guidelines [46]. DTT pathogens were also associated with larger defect sizes and greater PerOssal^®^ volume usage, although this effect remained non-significant. As the present study lacked a control group for evaluating alternative therapeutic approaches in DTT cases, it remains unclear whether PerOssal^®^ is particularly limited in treating DTT pathogens or if it performs better compared to other strategies within this subgroup. Kaplan–Meier analysis revealed a median time to revision surgery of 69 days and a mean time of 116 days, suggesting an adequate minimum follow-up duration of 180 days, with an average follow-up period of 2.2 years (see Table 3). The Kaplan–Meier curve displayed two distinct drops (see Figure 3c): the first appeared to correlate with early wound healing disorders, and the second with later reinfections (see Figure 3d). Notably, the number of revision surgeries increased when the first revision surgery was delayed, possibly indicating that an early, thorough revision may prevent subsequent surgeries (see Figure 3e).

The analysis of the osseous integration of PerOssal^®^ and the consolidation of nonunions revealed an overall failure rate of 11% (see Figure 4a), which decreased to only 8.5% in cavitary defects, resulting in a successful integration in 91.5% of cases involving cavitary defects but only a 72.7% consolidation of nonunions in segmental defects (see Figure 4b). Reinfection significantly increased the failure rate to 37.5%, indicating the critical need for high antibiotic efficacy.

### 4.2. Comparison to Other PerOssal^®^-Studies

Two other studies utilizing PerOssal^®^ in relevant patient cohorts have been published to date [35,37]. Sambri et al. examined a cohort of 93 patients [37] while Visani et al. investigated 52 patients [35]. Both cohorts comprised noticeably younger patients, with mean ages of 40 and 36 years, respectively, compared to the cohort in the present study which had a mean age of 54 years. Additionally, 98.6% of patients in the present presented with a Cierny–Mader anatomic type III or IV disease, which is associated with higher reinfection rates compared to anatomic type I diseases [37]. The patient cohort in this study therefore differs significantly from the cohorts studied by Sambri et al. and Visani et al., where only 52.7% and 31.9% of patients, respectively, had a Cierny–Mader anatomic type III or IV disease.

Visani et al. also assessed segmental bone defects (8%) similarly to the present study (13.4%), whereas Sambri et al. focused solely on cavitary bone defects. When reported, the localization of defects was similar. Sambri et al. reported 81.7% of defects in the femur and tibia, compared to 65.9% in this study, where an additional 20.7% were located in the calcaneus. Visani et al. did not provide specific localization data but stated that the lower limb was the predominant site of infection and treatment.

Sambri et al. specifically excluded patients with chronic kidney disease from their analysis. Notably, these patients exhibited a significantly higher incidence of reinfection (Figure 2b) and wound healing disorders than in the present study. The finding aligns with the existing literature, which identifies patients with chronic kidney disease as having increased infection risks and impaired wound healing [47,48].

As both studies were conducted in Italy, a higher prevalence of MRSA was expected according to global prevalence analyses [49]. This is consistent with the 29.5% MRSA rate reported by Sambri et al., compared to a significantly lower rate of only 2.6% in the present study. Visani et al. identified *Staphylococcus aureus* species in 53.7% of all culture-positive cases but did not differentiate between MRSA and MSSA. Accordingly, Vancomycin-loading was the predominant antibiotic treatment in either of the aforementioned studies (Visani et al. 100%, Sambri et al. 86%), whereas Gentamicin was the primary antibiotic used in 69.5% of cases in this study, with Vancomycin added in 11% of cases. Similar to the findings in the present study, Sambri et al. reported no correlation between culture-positive microbiological testing and reinfection, indicating a robust and effective antibiotic treatment strategy with PerOssal^®^. No specific information on a difficult-to-treat (DTT) pathogen subgroup among culture-positive patients was provided in other studies, which prevents a direct comparison with the trend observed in the present study, where a higher mean number of revision surgeries was found in the DTT pathogen subgroup (44.7% of patients with culture-positive microbiological results). Culture-negative microbiological results were comparable, with 46% reported by Visani et al., 34.4% by Sambri et al., and 53.7% in the present study.

The overall reinfection rate in the present study for a population with segmental and cavitary bone defects was 19.5%, compared to 13.5% in the work of Visani et al. This discrepancy may be attributed to the aforementioned differences in anatomic types of osteomyelitis and the significantly younger patient cohort. In the present study, an increase in comorbidities in older patients was observed, resulting in a shift towards a Cierny–Mader Class B host population, which is known to have higher reinfection rates than Class A host individuals [37,50].

The reinfection rate for cavitary defects, which were all cases of COM in the present study, was 20.0%, comparable to the 22.6% reported by Sambri et al. This finding was surprising given the differences in patient age (mean age difference of 14 years), anatomic type according to the Cierny–Mader classification, and the exclusion of patients with chronic kidney disease. The mean follow up was similar, with 21 months reported by Sambri et al. and 26.4 months in the present study. However, the minimum follow-up period in the study of Sambri et al. was 12 months, compared to 6 months in the present study, which may have masked reinfections in the older and more comorbid patient cohort analyzed in the present study.

### 4.3. PerOssal^®^ Compared to Other Ceramic Bone Graft Substitutes

A variety of other ceramic bone graft substitutes are currently available on the market [51]. Among these, a subset can also be loaded with antibiotics. The first product of this kind was Osteoset^®^-T (Wright medical group) [52], followed by Cerament^®^ (Bonesupport AB) a few years later [23,53,54,55].

Studies on Cerament^®^ report low reinfection rates of only 4% in cases of COM. Notably, the initial study included only small segmental defects of less than 1 cm. The general size of cavitary bone defects was not reported [23]. More recent promising data show similarly low reinfection rates of 4.4% in defects with a mean volume of 10.9 cm^3^ [53]. However, other studies have reported higher rates of persistent COM reaching 10%, with revision rates reaching 50%, primarily due to wound drainage [54]. Compared to PerOssal^®^, in the present study reinfection rates were higher (19.5% vs. 4–10%). Revision rates are challenging to compare due to the high variability in the publicly available literature (PerOssal^®^ 32.9% vs. Cerament^®^ 4–50%).

One possible explanation for the discrepancy in revision rates between PerOssal^®^ and Cerament^®^ may be the difference in preparation techniques. PerOssal^®^ is applied as a dry pellet, whereas Cerament^®^ is mixed intraoperatively and molded to fit the defect. This approach may enhance the bone-graft interface but could also contribute to a higher incidence and prolonged duration of wound drainage [23,54]. Nevertheless, recent data suggest that prolonged wound drainage in patients treated with Cerament^®^ might be a transient issue that does not indicate reinfection or the need for revision surgery [53].

Osteoset^®^-T is particularly noteworthy by comparison, as it is also provided in the form of pre-molded, resorbable beads. It has been examined in three large cohorts, each comprising over 100 patients [53,56,57]. The patient cohorts in which Osteoset^®^ T was used were slightly younger compared to the present cohort (46 and 47.4 years vs. 54 years). The severity of COM was similar, with a high percentage of Cierny–Mader type III and IV COM. Notably, reinfection was observed in only 9.2% and 11.2% of cases compared to 19.5% in the PerOssal^®^ cohort [53,57].

Fergusson et al. directly compared Cerament^®^ and Osteoset^®^-T in their study and concluded that improved bone healing and reduced infection recurrence occurred when Cerament^®^ was used [53]. However, a direct comparison with PerOssal^®^ remains difficult due to the differences in patient cohorts. Further studies are necessary to comprehensively evaluate the benefits and limitations of PerOssal^®^ and Cerament^®^.

### 4.4. Functional Results After the Use of PerOssal^®^ in Context

To our knowledge, no other studies have been conducted so far that investigate the functional results measured by the Lower Extremity Functional Scale (LEFS) following the use of PerOssal^®^ or other ceramic bone graft substitutes. In addition, no patient-reported outcome measures (PROMs) have been published on the use of PerOssal^®^ at all. Therefore, the results presented in this study, with a mean LEFS score of 53.4 (see Figure 1a), are particularly noteworthy in the context of the limited existing literature on this topic. While other PROMs, such as the American Orthopedic Foot and Ankle Society (AOFAS) Ankle-Hindfoot Score [58], the Disabilities of the Arm, Shoulder and Hand (DASH) Score [59], and the Short-Form Health Survey 36 item Score (SF-36) [54] are published after the use of Cerament^®^, direct comparison to the LEFS Score would not be suitable.

In the present study, no significant differences were observed in outcomes based on localization. Similarly, no correlation was found between the follow-up period and LEFS scores, suggesting that further improvements are unlikely with extended follow-up or later measurements of the LEFS in the future. However, women demonstrated a markedly superior outcome compared to men. Notably, the female cohort was younger and had fewer comorbidities, both of which are known to influence LEFS [60,61,62] and other PROMs [63] in COM.

The mean LEFS score in an uninjured population is 77 [39,64], indicating that even in patients not suffering from reinfection in our cohort, there remains a significant functional impairment in patients treated for COM or NU with PerOssal^®^ (see Figure 1b). This is in line with other prior studies which have already presented functional outcomes with the LEFS in patients who have undergone treatment for COM. In their study, Campbell and colleagues reported the treatment of 12 patients with a predominantly staged skeletal stabilization approach. The analyzed cohort was younger than the cohort in the present study (39 vs. 54 years) but the mean number of procedures undergone by patients prior to the study procedure was higher (8.4 vs. 7.3). In Campbell et al. all patients needed flap coverage treatment, compared to 34.1% in the present study. Mean LEFS score was 51 points at the 4.4 year follow-up [65].

Wu et al. [66] described the treatment of Cierny–Mader Type IV COM with a two-stage induced membrane technique and the filling of the bone void with either pure autologous bone graft material or a mixture of autograft and allograft material. The patients in the study by Wu et al. were, on average, 13 years younger than those in the present study (mean age: 41 vs. 54 years), had a significantly shorter duration of infection (2.5 years vs. 8.9 years), and had undergone fewer infection-related surgeries (2.9 vs. 5.0). The differences make it challenging to directly compare outcome parameters between the two cohorts. However, the cohort in Wu et al. had a mean LEFS score of 65.6 points after a 2.5 year follow-up period and an infection cure rate of 97%.

The data of the current study show a proportional relationship between the number of infection-related previous surgeries and the probability of requiring soft tissue coverage with a free flap. The number of infection-related previous surgeries was also inversely proportional to the LEFS Score. This phenomenon could partially account for the discrepancy observed between the findings of Wu, those of Campbell et al., and the present study. In the future, bioactive adjuvants like parathyroid hormone could further enhance the possibilities of ceramic-based bone grafts [26]. Nevertheless, both the existing literature and the results of this study indicate improved outcomes in patients treated with PerOssal^®^, supporting the broader adoption of this approach. These findings should encourage practitioners and patients to prioritize early treatment of chronic osteomyelitis (COM) and nonunion (NU) using this method to optimize clinical and functional outcomes.

### 4.5. Limitations

As stated above, the parameters of revision surgery, reinfection, and osseous integration are interrelated factors, making it challenging to determine the independent effect of each. The cohort in this study primarily includes patients with cavitary defects, so the findings are most applicable to this specific group. With a minimum follow-up time of only six months, reinfection rates could potentially be underestimated, as both Sambri et al. and the present data show that reinfections can occur later after surgery. However, the mean follow-up duration in the present study of 2.2 years significantly exceeds the latest observed reinfection, which occurred at approximately 1.5 years post-surgery. Furthermore, the median time to reinfection was 69 days or 2.3 months, compared to 11 months in Sambri et al., suggesting that the majority of reinfections are likely accounted for within our follow-up period. Although a variety of pre-existing conditions were monitored, obesity was not included as a potential confounder, which limits the conclusions that can be drawn about its impact in this patient cohort. However, high blood pressure and diabetes mellitus—key components of metabolic syndrome—were analyzed, providing indirect insights into the potential influence that obesity could have on outcomes. Osseous integration of PerOssal^®^ was assessed solely through biplanar postoperative X-rays rather than CT scans. Furthermore, no specific scoring system, such as the Goldberg Score [67], was utilized, which may have limited the precision of these assessments. Despite this limitation, the observer was fully blinded, and multiple X-ray views were evaluated for each patient, reducing the potential for subjective bias. Finally, as a retrospective study, there are inherent limitations, including potential selection and information bias associated with the patient cohort examined.

## 5. Conclusions

The current study represents the second-largest cohort to date analyzing the use of PerOssal^®^ in the treatment of chronic osteomyelitis and nonunions and is the only study measuring patient-reported outcomes. The findings highlight the complexity of managing advanced cases, particularly those with Cierny–Mader type III and IV classifications, challenging microbiological profiles, and prolonged infection histories. Despite the older age and higher comorbidity burden of this cohort compared to most comparable studies, promising infection eradication rates exceeding 80% were observed. Functional impairments, as measured by LEFS scores, and higher numbers of revision surgeries in cases with difficult-to-treat pathogens emphasize the need for critical patient selection, as defect characteristics and comorbidities play essential roles in determining outcomes. Further studies are warranted to refine treatment strategies and enhance functional recovery.

## Figures and Tables

**Table 1:** Inclusion and exclusion criteria.

Inclusion	Exclusion
Treatment of NU or COM with PerOssal^®^ between January 2020 and December 2023	Age < 18 years
	Inability to provide written consent
	Cierny–Mader Type C host

**Table 2:** Collected Parameters.

Parameter Group	Parameter [Unit]
Patient characteristics	Age [years] Sex [male/female] American Society of Anesthesiologists (ASA)-score Past medical history, Diagnosis (COM, SNU or ANU), Date of initial trauma (if applicable) Number of previous surgeries due to trauma (if applicable) Date of first mention of COM or NU Number of previous surgeries due to COM or NU Prior positive microbiological testing
Index surgery	Date of index-surgery (PerOssal^®^) Localization Defect size extracted from biplanar X-ray images Defect characteristics (cavitary vs. segmental) Classification of COM after Cierny–Mader Plastic soft tissue coverage Microbiological testing in index-surgery, Difficult to treat pathogen ^1^ Orthopedic implant Used volume of PerOssal^®^ One-step or two-step protocol Antibiotic loading of PerOssal^®^ Additional allo- or autograft
Outcome measures	Allergies, Reinfection or persistent NU after index surgery Implant failure Revision surgery [yes/no] Number of revision surgeries Integration of PerOssal^®^ after index surgery Consolidation period in segmental defects or period of integration of PerOssal^®^ in cavitary defects LEFS

^1^ See Section 3.1 for details.

**Table 3:** Patient characteristics. ANU: Aseptic Nonunion. ASA: American Society of Anesthesiologists. COM: Chronic Osteomyelitis. SNU: Septic Nonunion. * = *p* < 0.05 Age_Male_ vs. Age_Female_.

Patient Characteristics		Frequency	Percent [%]	Mean ± SEM
Sex	Male	65	79.3	
	Female	17	20.7	
Age [years]	Total			54.3 ± 1.7
	Male			56.0 ± 1.9 *
	Female			47.5 ± 3.8 *
Diagnosis	COM	70	85.4	
	SNU	6	7.3	
	ANU	6	7.3	
Defect Size [cm]				4.72 ± 0.38
PerOssal Volume	1	1	1.2	
[N_beads_]	12	25	30.5	
	24	10	12.2	
	36	1	1.2	
	50	38	46.3	
	100	7	8.5	
ASA-Score	1	8	9.8	
	2	40	48.8	
	3	30	36.6	
	4	4	4.9	
Localization	Humerus	5	6.1	
	Radius	1	1.2	
	Pelvis	2	2.4	
	Femur	19	23.2	
	Tibia	35	42.7	
	Fibula	2	2.4	
	Calcaneus	17	20.7	
	Forefoot	1	1.2	
Cierny–Mader anatomic type	I	1	1.4	
	II	0	0	
	III	32	45.7	
	IV	37	52.9	
Antibiotic loading	Vancomycin	17	20.7	
	Gentamicin	48	58.5	
	Vancomycin + Gentamicin	9	11.0	
	Others	8	9.8	
Flap coverage	Yes	28	34.1	
	No	54	65.9	
Fracture to PerOssal [years]				13.9 ± 2.0
Infection to PerOssal ^1^ [years]				8.9 ± 1.7
Follow up [years]				2.2 ± 0.1
Previous surgeries	Non-infection related			2.3 ± 0.4
	Infection related			5.0 ± 0.7

^1^ if applicable.

**Table 4:** Pathogens at index surgery.

Pathogen Group	Pathogen	Frequency	Percent [%]
Gram-positive ^1^	*Staph. aureus (MSSA)*	12	31.6
	*Staph. aureus (MRSA)*	1	2.6
	*Staph. epidermidis*	3	7.9
	Other Staphylococci	3	7.9
	*Enterococcus* spp.	3	7.9
	*Corynebacterium* spp.	5	13.2
	*Cutibacterium* spp.	2	5.3
	*Actinomyces* spp.	2	5.3
Gram-negative ^1^	*E. coli*	3	7.9
	*Pseudomonas* spp.	8	21.7
	*Proteus* spp.	4	10.5
	*Enterobacter* spp.	6	15.8
	*Bacteroides fragilis* spp.	2	5.3
	*Fusobacterium* spp.	2	5.3
	*Klebsiella* spp.	2	5.3
*Candida* spp. ^1^		2	5.3
Multidrug-resistant organisms (MDRO) ^1,3^		16 in 14 patients	36.8
Difficult-to-treat organisms ^1,4^		19 in 17 patients	44.7
Polymicrobial infection ^1,5^		20	52.6
Culture-negative infection ^2^		44	53.7

MSSA: methicillin-sensitive *Staphylococcus aureus*; MRSA: methicillin-*resistant Staphylococcus aureus*. ^1^ Percentage indicates fraction of patient cohort with a culture-positive infection. ^2^ Percentage indicates fraction of total patient cohort. ^3^ Common multidrug-resistant organisms (MDRO) include vancomycin-resistant Enterococci (VRE), methicillin-resistant *Staphylococcus aureus* (MRSA), and multidrug-resistant Gram-negative bacteria (MDRGN). ^4^ Difficult-to-treat organisms: *Enterococcus* spp., MDRGN, MRSA, and *Candida* spp. ^5^ Polymicrobial infection was defined by more than one detected germ.

## Data Availability

The data presented in this study are available on request from the corresponding author due to privacy or ethical restrictions.

## Supplementary Materials

The following supporting information can be downloaded at: https://www.mdpi.com/article/10.3390/jcm13247764/s1, Figure S1: Additional analysis of reinfection; Figure S2: Biplanar X-rays of the tibia at three different time points following the index surgery.

## Abbreviations

ANOVA	Analysis of Variance
ANU	Aseptic Nonunion
ASA	American Society of Anesthesiologists
CKD	Chronic Kidney Disease
COM	Chronic Osteomyelitis
DTT	Difficult-to-Treat
LEFS	Lower Extremity Functional Scale
MCID	Minimal Clinically Important Difference
MDRO	Multidrug-Resistant Organisms
MDRGN	Multidrug-Resistant Gram-Negative
MRSA	Methicillin-Resistant Staphylococcus aureus
MSSA	Methicillin-Sensitive Staphylococcus aureus
NU	Nonunion
OM	Osteomyelitis
PMMA	Polymethylmethacrylate
SNU	Septic Nonunion
VRE	Vancomycin-Resistant Enterococci
WHD	Wound Healing Disorder
