# Supplementary material for: Treatment of High-Grade Chronic Osteomyelitis and Nonunions with PerOssal®: A Retrospective Analysis of Clinical Efficacy and Patient Perspectives
Source: J Clin Med. 2024 Dec 19;13(24):7764. doi: 10.3390/jcm13247764 (PMC11727840; doi:10.3390/jcm13247764)
Supplement: Supplementary file 1 [file jcm-13-07764-s001.zip › jcm-3352077-supplementary.pdf]

Journal of
Clinical Medicine

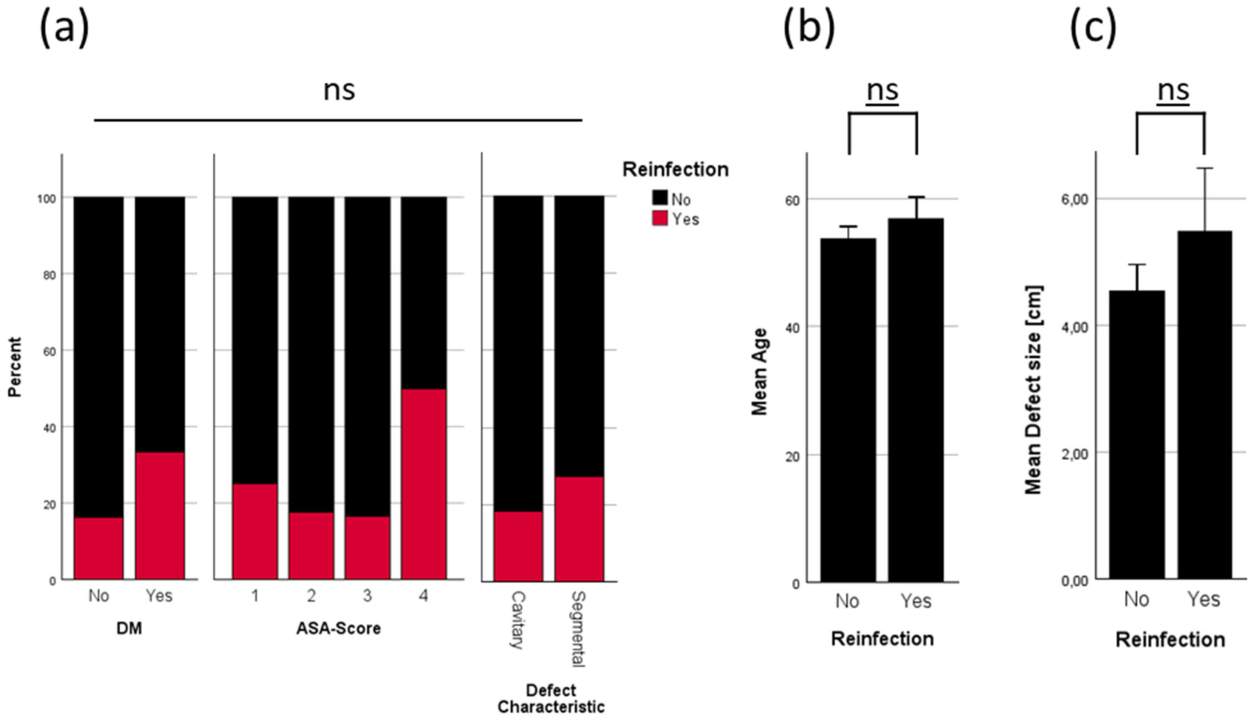

Figure S1. Additional analysis of reinfection: (**a**) Influence of DM, ASA-Score, and defect characteristic on reinfection rate; (**b**) Age of patients without and with reinfection; (**c**) Defect size without and with reinfection. DM: Diabetes Mellitus, ASA-Score: American Society of Anesthesiologists - score, Ns = not significant.

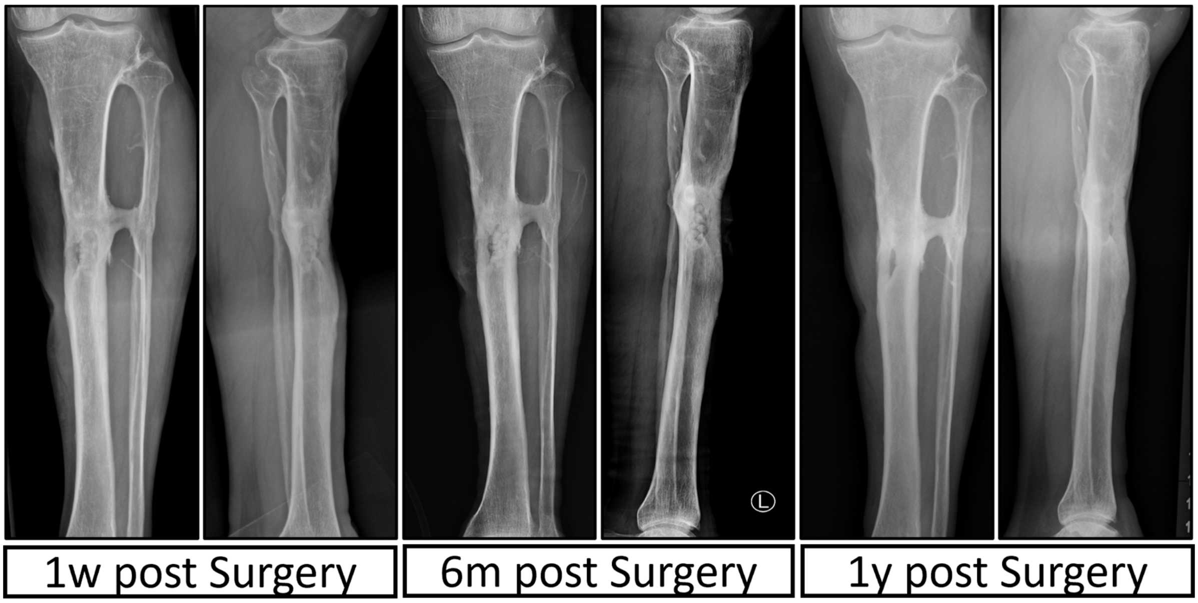

Figure S2. Biplanar X-rays of the tibia at three different time points following the index surgery. PerOssal is clearly visible in the first two images but begins to blend with the surrounding bone at 6 months post-surgery and demonstrates complete integration by 1 year.
